# Supplementary figures and images for: Bacterial behavior in human blood reveals complement evaders with some persister-like features
Source: PLoS Pathog. 2020 Dec 16;16(12):e1008893. doi: 10.1371/journal.ppat.1008893 (PMC7773416; doi:10.1371/journal.ppat.1008893)

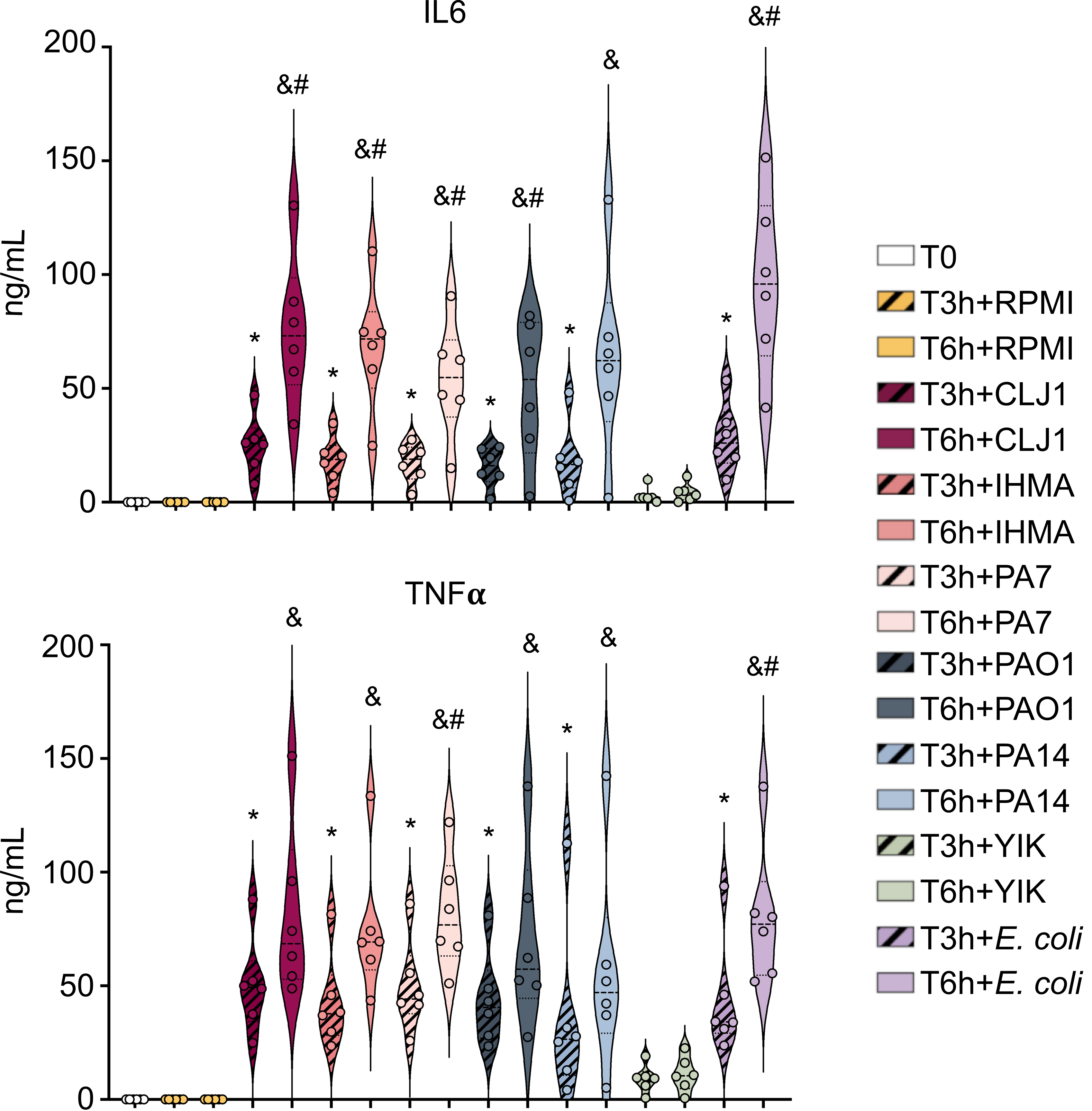

Supplement: S1 Fig — Strains, as indicated, were incubated in HWB (n = 6, from different donors) for the time points indicated, and cytokines were quantified by a multiplex analysis. (*): different from T3h+YIK. (&): different from T6h+YIK. (#): different from the cognate T3h. Mann-Whitney test: # p <0.05. Kruskal-Wallis test, p <0.05; Student-Newman-Keuls post-hoc test: * and &, p <0.05. (TIF) [file ppat.1008893.s001.tif]

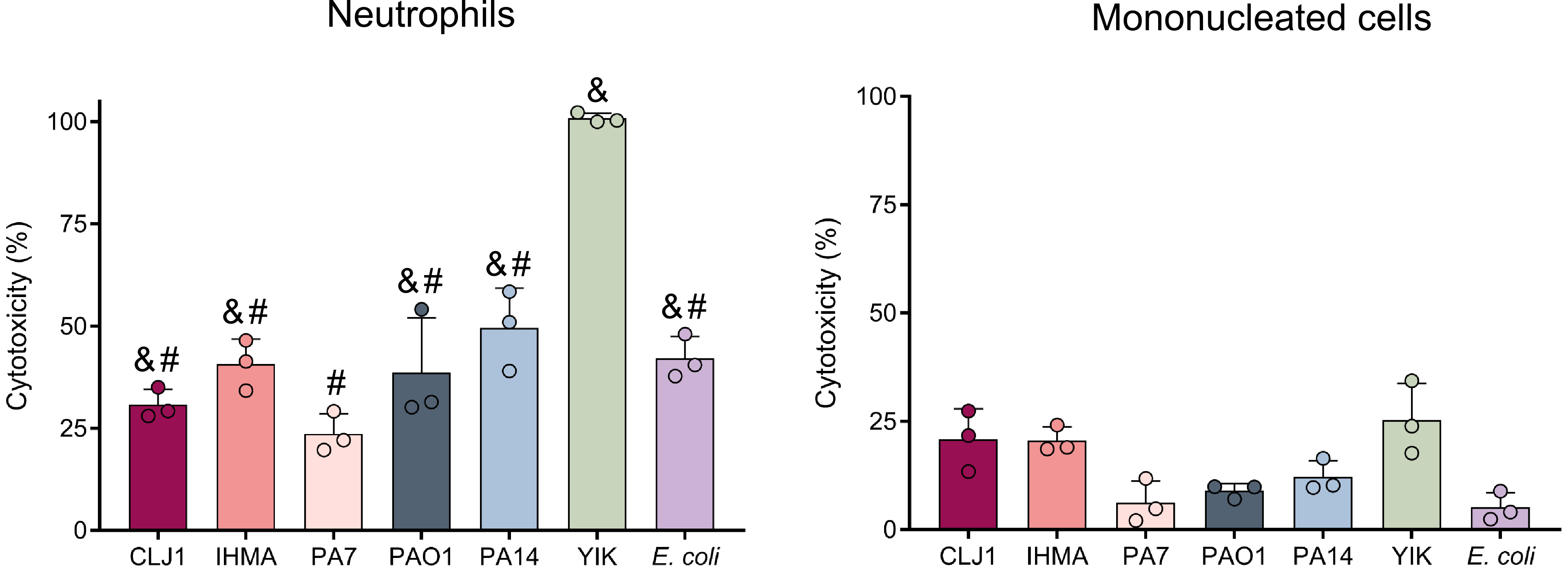

Supplement: S2 Fig — Cytotoxicity of indicated strains on purified neutrophils or mononucleated cells was analyzed by LDH release (n = 3, from different donors). Following a 3-h incubation of leukocytes with bacteria at a MOI of 5, LDH released by dead cells was quantified from the supernatants. The LDH release obtained following treatment with 1%Triton X-100 correspond to 100% cytotoxicity. (&): different from PA7. (#): different from YIK. Kruskal-Wallis test, p <0.05; Student-Newman-Keuls post-hoc test: & and #, p <0.05. Note that none of the strains tested induced a significantly different killing of mononucleated cells. (TIF) [file ppat.1008893.s002.tif]

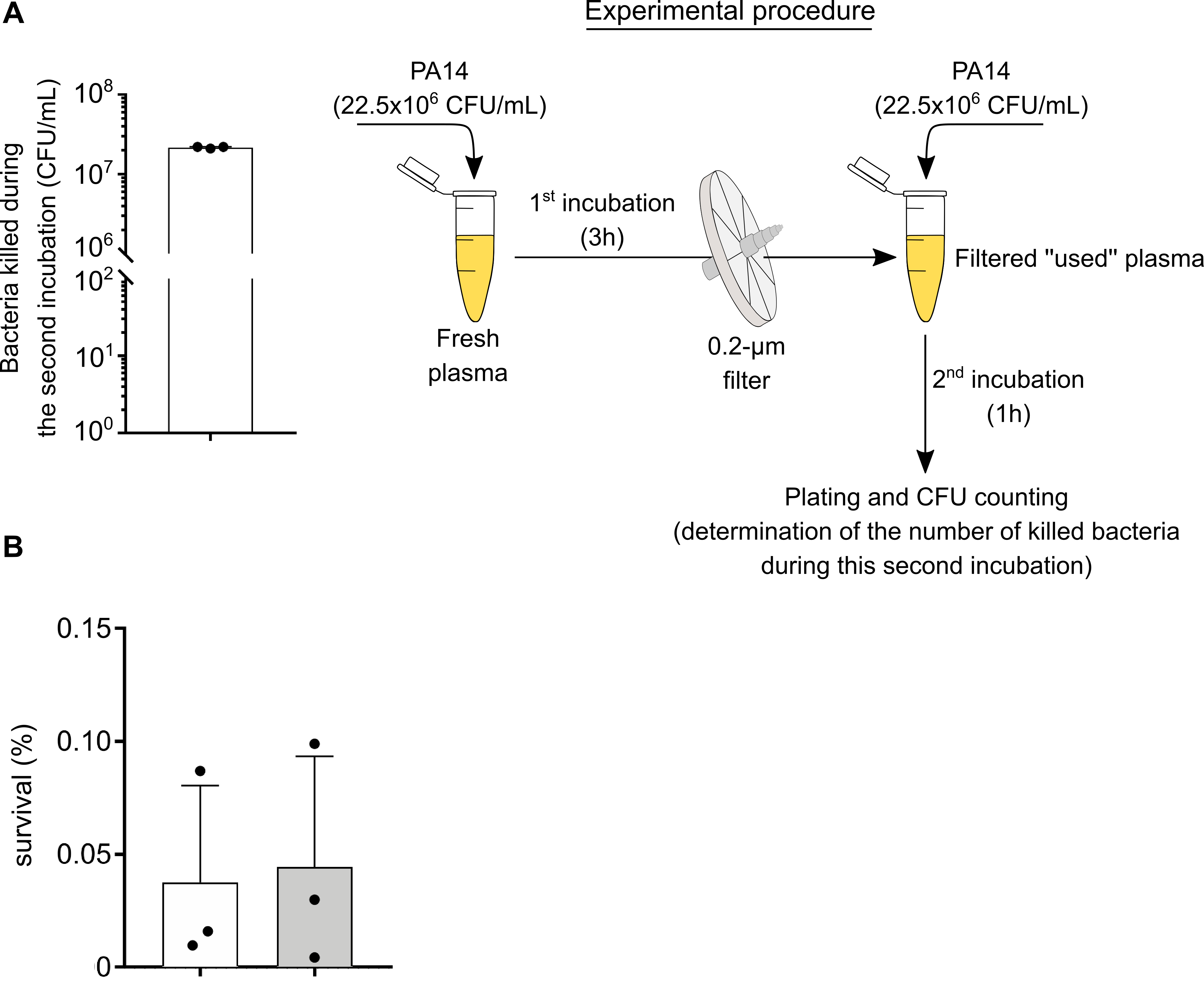

Supplement: S3 Fig — (A) Following a first challenge, the plasma is still bactericidal. Schematic view of the experimental procedure: PA14 (22.5x106 CFU/mL) was incubated in a pool of plasma for 3 h. Following the first challenge, the plasma was sterilized by passing through a 0.2-μm filter, and re-challenged with the same number of PA14 cells for 1 h at 37°C. More than 20x106 bacteria/mL were eliminated during this second incubation, showing that the plasma is still bactericidal after 3 h of contact with the bacteria. (B) Reducing the bacterial load does not affect the proportion of surviving cells. PA14 was incubated in a pool of plasma at concentrations of 22.5x106 (white) or 2.25x10^6^ (grey) CFU/mL, and survival was assessed following incubation for 3 h. The proportions of evaders were unchanged regardless of the initial bacterial load. Data represent mean ± SD of three independent experiments (A and B). (TIF) [file ppat.1008893.s003.tif]

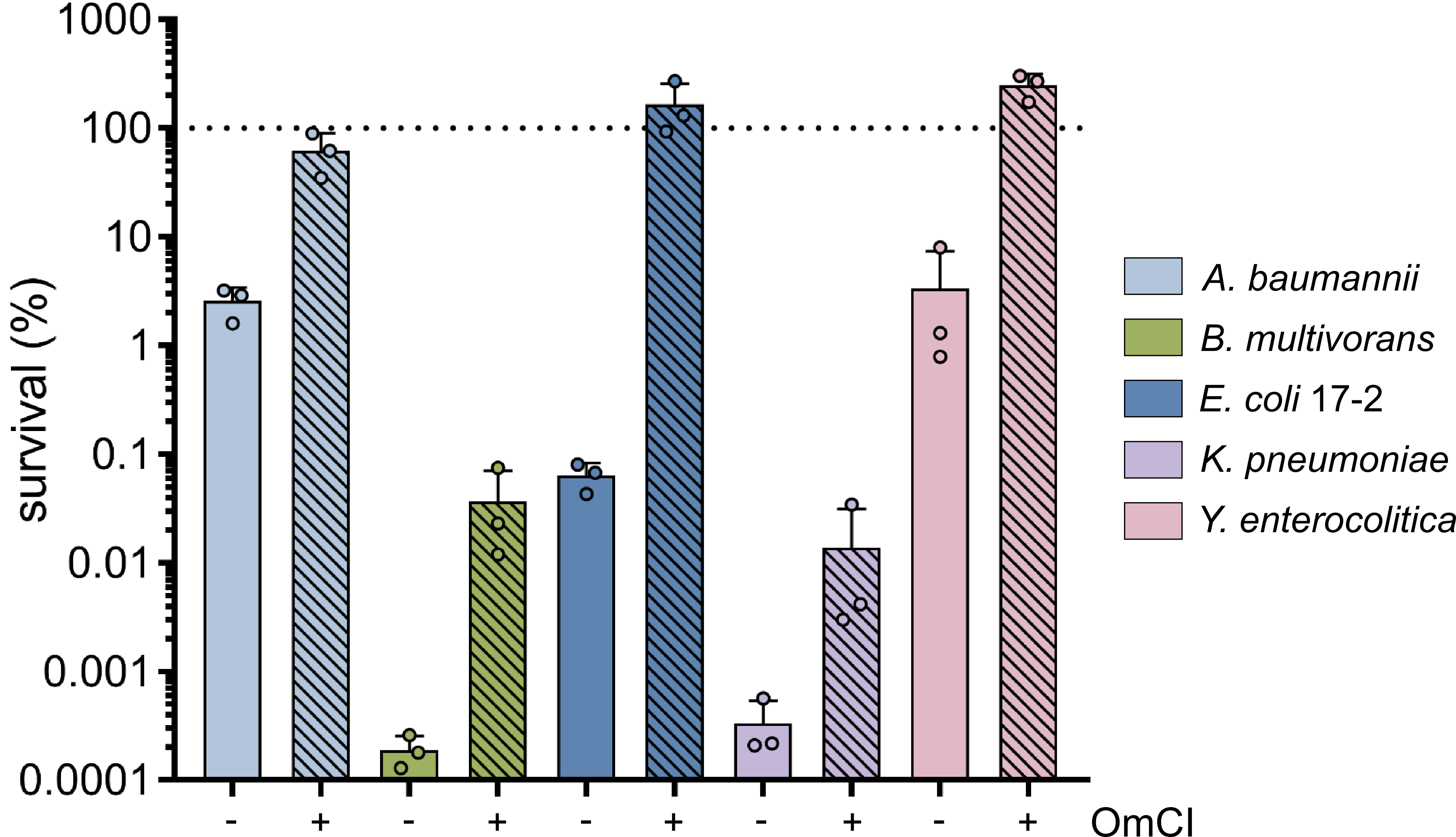

Supplement: S4 Fig — Depending on the species, additional fluid-phase bactericidal effectors other than complement are likely involved in the selection of evaders. Bacteria were incubated in a pool of human plasma for 3 h with (hashed) or without (solid) OmCI at 20 μg/mL (A. baumannii and Y. enterocolitica) or 40 μg/mL (B. multivorans, E. coli 17–2 and Y. enterocolitica) and survival was measured based on CFU counts. Data represent mean ± SD of three independent experiments. (TIF) [file ppat.1008893.s004.tif]

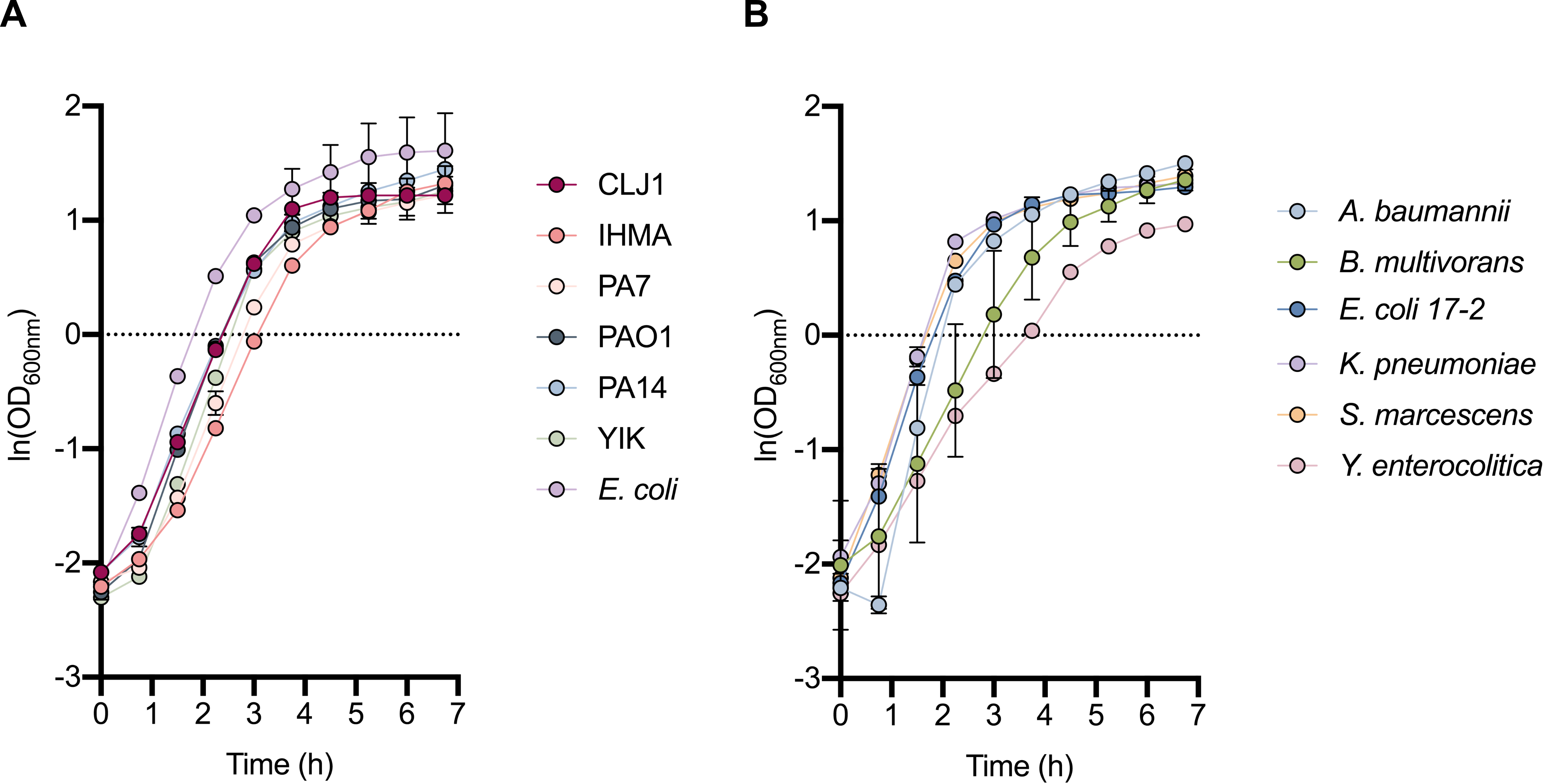

Supplement: S5 Fig — Growth kinetics in LB of the six P. aeruginosa strains CLJ1, IHMA, PA7, PAO1, PA14, YIK, E. coli CF7968 (A), and the other Gram-negative species used in this study (B). Data represent mean ± SD of two independent cultures (A and B). (TIF) [file ppat.1008893.s005.tif]

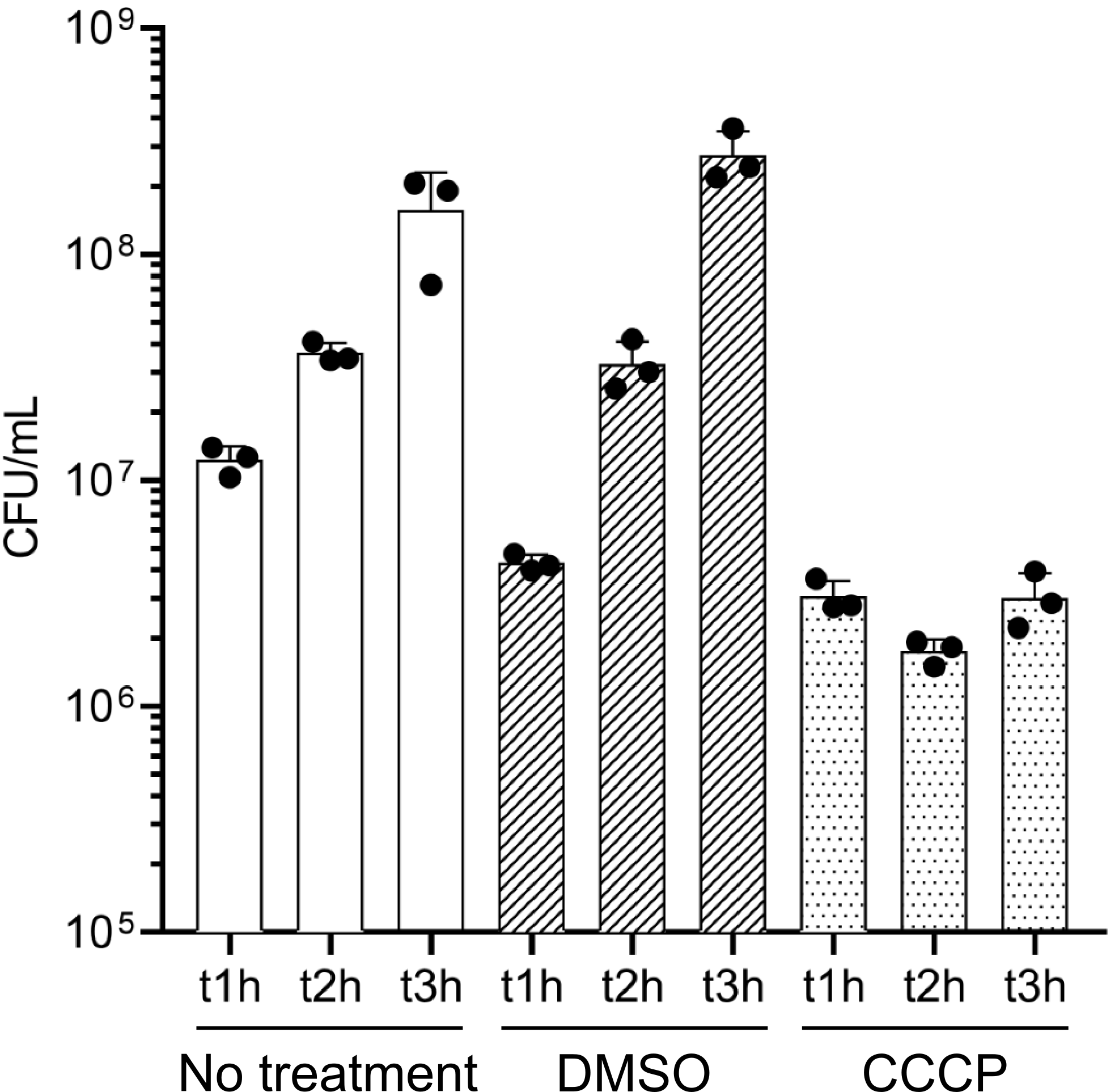

Supplement: S6 Fig — Exponentially growing IHMA87 cultures in LB were either not treated (solid), treated 1 h with CCCP at 200 μg/mL (dotted) or exposed to 0.5% DMSO for 1 h (hashed). Cells were subsequently recovered and incubated in LB alone. Evolution of the CFU number was monitored by plating after 1 h, 2 h and 3 h. Bacterial survival is unaffected following CCCP treatment, however treated cells are still non-growing even 3 h after removing the stress. At 0.5%, DMSO does not affect bacterial growth. Data represent mean ± SD of three independent experiments. (TIF) [file ppat.1008893.s006.tif]

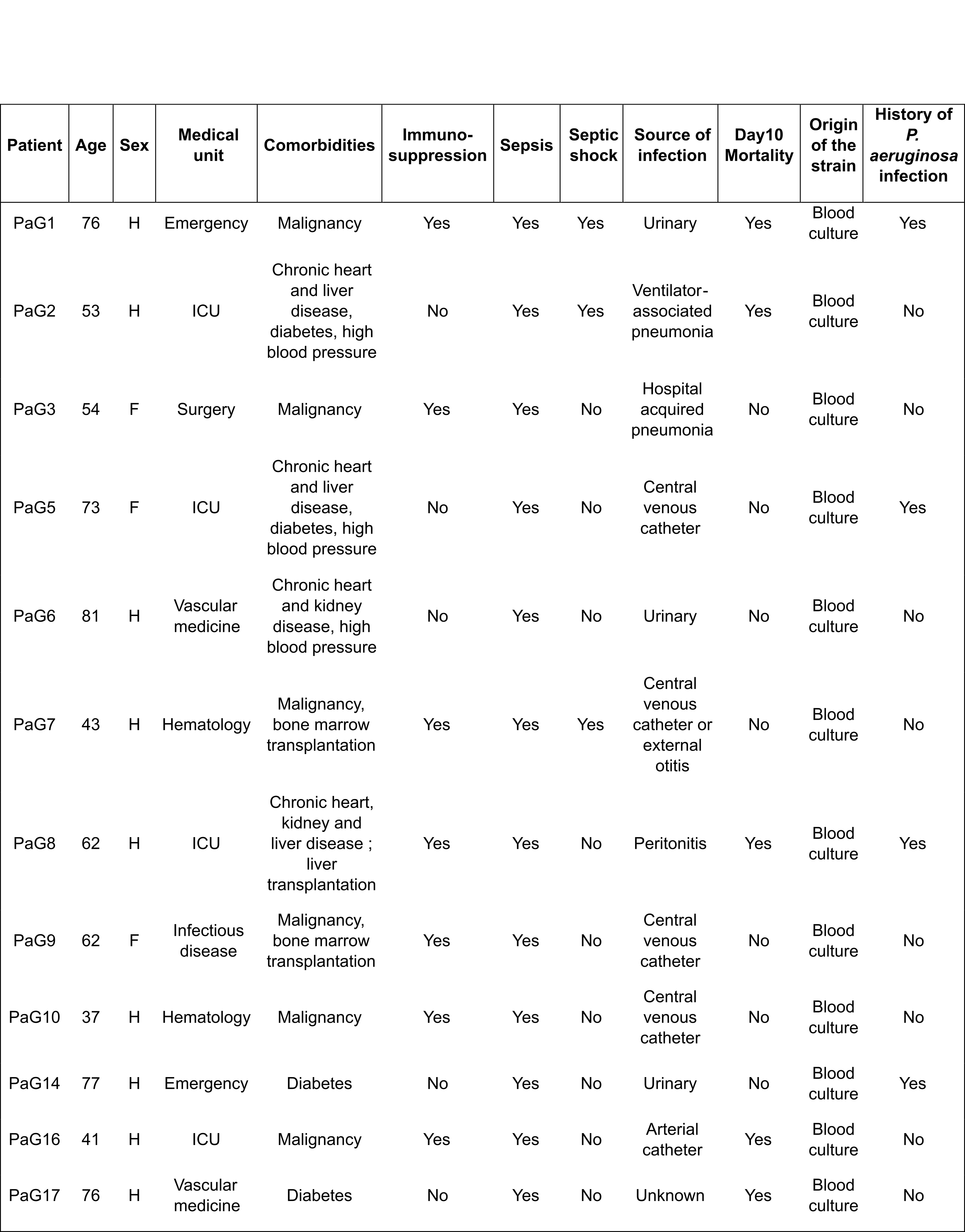

Supplement: S2 Table — (TIF) [file ppat.1008893.s008.tif]
