# Supplementary material for: Bacterial behavior in human blood reveals complement evaders with some persister-like features
Source: PLoS Pathog. 2020 Dec 16;16(12):e1008893. doi: 10.1371/journal.ppat.1008893 (PMC7773416; doi:10.1371/journal.ppat.1008893)
Supplement: S1 Table — (PDF) [file ppat.1008893.s007.pdf]

Strains	Characteristics/Source	Reference/Origin
P. aeruginosa		
PAO1	TTSS$^+$, ExoS$^+$, O5, Wound	Holloway 1955, lab collection
PA14	TTSS$^+$, ExoU$^+$, O10, Burn	Rahme *et al.* 1995, Lory lab
PA7	ExlA$^+$, O12, Burn (taxonomic outlier)	Roy *et al.* 2010
CLJ1	ExlA$^+$, O12, Hemorrhagic pneumonia	Elsen *et al.* 2014
IHMA879472 (IHMA87)	ExlA$^+$, O11+O12, Urinary tract	Kos *et al.* 2015, IHMA*
YIK	TTSS$^+$, ExoU$^+$, O8, Bacteremia	Ellabadi *et al.* 2019
IHMA87-GFP	IHMA87 with constitutive expression of GFP, carrying miniCTX-*P*X2-GFP	This study
***P. aeruginosa* BSI isolates**		
PaG1	O8, Infected organ	This study/Grenoble Hospital
PaG2	O5, “	“
PaG3	O6, Contaminated catheter	“
PaG5	O4, “	“
PaG6	O6, Infected organ	“
PaG7	O1, Contaminated catheter	“
PaG8	O4+O10, Infected organ	“
PaG9	O10, Contaminated catheter	“
PaG10	O6, “	“
PaG14	O4, Infected organ	“
PaG16	O2, Contaminated catheter	“
PaG17	O3, Infected organ	“
**Other Gram-negative species**		
Acinetobacter baumannii ATCC23220	-	Thornley 1967, lab collection
Burkolderia multivorans ATCC17616	-	Stanier *et al.* 1966, lab collection
Escherichia coli CF7968	MG1655 corrected to *rph*$^+$ and deleted for *lacIZ*	Hirsh, *et al.* 2002, D. Schneider lab
Enteroaggregative *Escherichia coli* 17-2	-	Aschtgen *et al*. 2008, E. Cascales lab
Klebsiella pneumoniae NCTC9633	-	Sneath *et al*. 1980, lab collection
Seratia marcescens Db10	-	Flyg *et al*. 1980, S. Coulthurst lab
Stenotrophomonas maltophilia ATCC13637	-	Hugh and Ryschenkow, 1961, lab collection
Yersinia enterocolitica MRS40	-	Sarker *et al*. 1998; G. Cornelis lab

*International Health Management Association (IHMA; USA).

Supplementary references

1. Thornley MJ. A taxonomic study of Acinetobacter and related genera. J Gen Microbiol 1967;49:211–57. https://doi.org/10.1099/00221287-49-2-211.
2. Stanier RY, Palleroni NJ, Doudoroff M. The aerobic pseudomonads: a taxonomic study. J Gen Microbiol 1966;43:159–271. https://doi.org/10.1099/00221287-43-2-159.
3. Aschtgen M-S, Bernard CS, De Bentzmann S, Lloubès R, Cascales E. SciN is an outer membrane lipoprotein required for type VI secretion in enteroaggregative Escherichia coli. J Bacteriol 2008;190:7523–31. https://doi.org/10.1128/JB.00945-08.
4. Sneath PHA, McGOWAN V, Skerman VBD. Approved Lists of Bacterial Names. Int J Syst Evol Microbiol 1980;30:225–420. https://doi.org/10.1099/00207713-30-1-225.
5. Flyg C, Kenne K, Boman HG. Insect pathogenic properties of Serratia marcescens: phage-resistant mutants with a decreased resistance to Cecropia immunity and a decreased virulence to Drosophila. J Gen Microbiol 1980;120:173–81. https://doi.org/10.1099/00221287-120-1-173.
6. Hugh R, Ryschenkow E. Pseudomonas maltophilia, an Alcaligenes-like Species. J Gen Microbiol 1961;26:123–32. https://doi.org/10.1099/00221287-26-1-123.
7. Sarker MR, Neyt C, Stainier I, Cornelis GR. The Yersinia Yop virulon: LcrV is required for extrusion of the translocators YopB and YopD. J Bacteriol 1998;180:1207–14.
