# Supplementary material for: Bacterial behavior in human blood reveals complement evaders with some persister-like features
Source: PLoS Pathog. 2020 Dec 16;16(12):e1008893. doi: 10.1371/journal.ppat.1008893 (PMC7773416; doi:10.1371/journal.ppat.1008893)
Supplement: Attachment — Submitted filename: Responses to reviewers Pont et al-.pdf [file ppat.1008893.s009.pdf]

Dear Dr Tang,

We would like to thank the editors and the reviewers for consideration of our manuscript. We have addressed their comments and provided additional data straightening our conclusions. Our responses to all points are below in italics.

Editor:
Thank you very much for submitting your manuscript "Bacterial behavior in human blood reveals complement evaders with persister-like features" for consideration at PLOS Pathogens. As with all papers reviewed by the journal, your manuscript was reviewed by members of the editorial board and by several independent reviewers. In light of the reviews (below this email), we would like to invite the resubmission of a significantly revised version that takes into account the reviewers' comments.
The reviewers raised a significant issue of whether the effects observed can be ascribed to evasion of complement rather than another heat labile mechanism of bacterial killing in plasma? They suggest some approaches to inhibit complement killing, and experiments could be performed with Mg2+/EGTA.

We provide here a significantly revised manuscript with three new experiments providing additional data on complement evasion. As suggested by one reviewer, we used the specific complement inhibitor OmCI, and assessed bacterial survival in its presence. The new results are presented in the main Figure 2 (for P. aeruginosa strains) and in Supplementary Figure 4 (for other species). The results of these experiments support our conclusion that the evaders resist the complement mediated killing. Accordingly, we modified the main text and cited the new references on the use of OmCI.

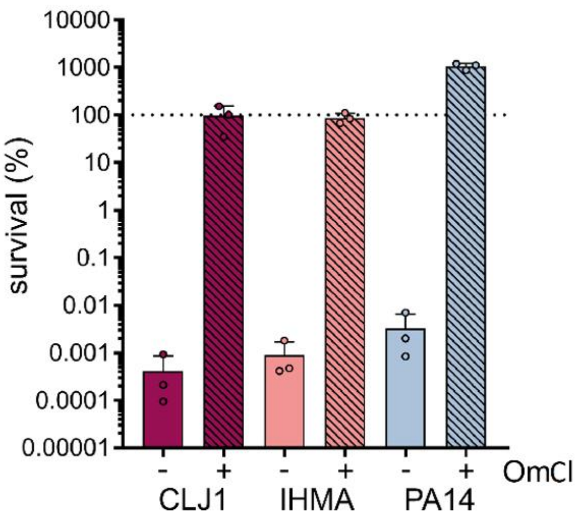

New Figure 2C: Inhibition of MAC formation rescues *P. aeruginosa* strains from killing. Bacteria were incubated in a pool of human plasma for 3 h with (hashed) or without (solid) OmCI at 20 µg/mL (IHMA87 and PA14) or 40 µg/mL (CLJ1) and survival was measured based on CFU counts. Due to bacterial clumping, sonication was used to increase the accuracy of CFU measurement of IHMA87, as described in Methods. Data represent mean ± SD of three independent experiments.

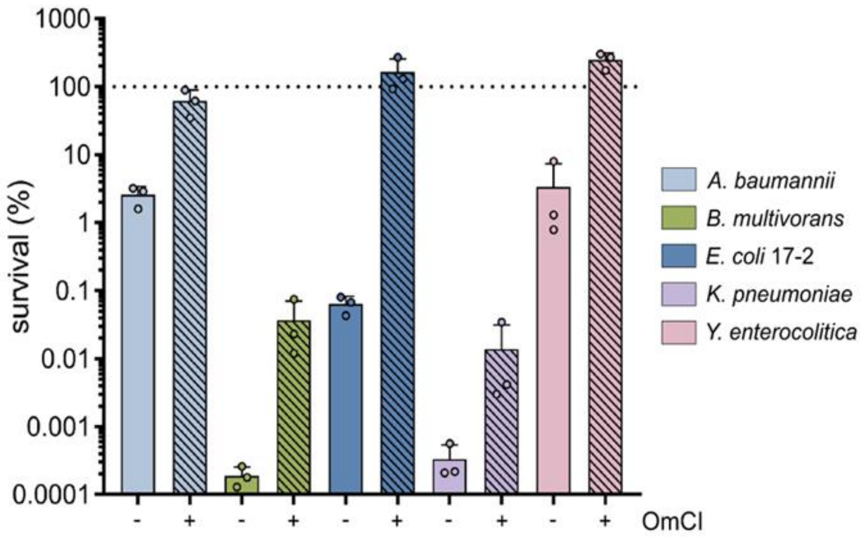

New Figure S4: Plasma evaders are either solely or partly composed of complement-tolerant cells. Depending on the species, additional fluid-phase bactericidal effectors other than complement are likely involved in the selection of evaders. Bacteria were incubated in a pool of human plasma for 3 h with (hashed) or without (solid) OmCI at 20 µg/mL (*A. baumannii* and *Y. enterocolitica*) or 40 µg/mL (*B. multivorans*, *E. coli* 17-2 and *Y. enterocolitica*) and survival was measured based on CFU counts. Data represent mean ± SD of three independent experiments.

Reviewer #1:

Part I - Summary

The paper describes a very interesting finding - the discovery of "evaders", phenotypic variants of bacterial cells that survive in blood. Most pathogen cells in blood are killed by a combination of complement and phagocytosis, but the authors of this study find that killing of P. aeruginosa and other pathogens in blood in biphasic, with a subpopulation of surviving evaders. They provide a good analysis of the obvious factors that may contribute to survival, such as the presence of toxins in the pathogens, but find that these are not involved in survival. Importantly, the authors show that this phenotypic subpopulation is different from persisters, since there are more of such cells in a growing population as compared to stationary (more persisters in stationary). Survival was also energy-dependent, suggesting the presence of an active process. It appears that variation in expression of some complement-protection factors confers resistance. This study sets the stage for identifying the mechanism of this interesting and important phenomenon. The study is well executed; experiments are simple and straightforward, with considerable statistical significance. Minor comments follow.

We thank to this reviewer for appreciation of our work.

Part II - Major issues: Key Experiments Required for Acceptance

None

Part III – Minor Issues: Editorial and Data Presentation Modifications

The abstract lacks any mechanistic information and does not do justice to this work. Please add information in the abstract related to specific factors examined in this study for their ability to contribute to survival, such as toxins and TIII secretion system.

We added the nature of the secreted toxins by the different isolates in the Abstract, line 32.

A very interesting isolate that was completely resistant to killing was not pursued. This is understandable, given the focus of the study on evaders, but it would be useful to note if there are known precedents of such isolates, and whether the mechanism for full resistance to blood has been described.

The reviewer is pointing to the Pa isolate YIK. Indeed, isolates of this type are rare and interesting. We only recently obtained a genome sequence of the YIK strain and are currently performing Tn-Seq in plasma to decipher underlying mechanisms of complement-resistance. In the blood, the complete survival is probably achieved through both complement resistance and cytotoxicity towards phagocytic cells as we observed in the new figure S2 for neutrophils.

There is a positive correlation between the level of survival of the bulk population and the level of evaders this population produces, makes sense to point this out.

Surviving cells of the bulk population (we guess this refers to Fig. 1A and 2A) represent the evaders, as survival is assessed at 3h, a time point which corresponds to the beginning of the ''survival plateau'' (Fig. 3A), at which only evaders are left. If the comment was made because the surviving cells could have been only partly composed of evaders, we hope our answer clarified the point.

Reviewer #2:

Part I - Summary

The manuscript of Pont et al., studies how P. aeruginosa can persist within the blood. With a panel of different P. aeruginosa strains it becomes clear that a small population survives in whole blood. This effect is also observed in plasma and can be heat inactivated. Since, complement is able to kill Gram-negative bacteria by forming pores in the membrane the surviving population is called 'complement evaders'. It is an interesting and relevant finding that a small part of the bacteria manages to survive in plasma. The complement evaders show sensitivity to plasma again after reexposure to plasma indicating that these are phenotypic variants. The authors evaluate if the complement evaders have similar characteristics as persisters. In contrast to persisters, the complement evaders are not more abundant in stationary phase cultures and experiments suggested that metabolic activity is required. The findings in this manuscript are important in the understanding how bacteria persist within the blood. The results will lead to follow-up studies for more detailed understanding how the described subpopulation survives in blood. The article would benefit from extra experiments that more definitely show that the bacteria indeed are persistent to complement. Plasma contains a lot of proteins that can have an effect on the survival of bacteria. Heat inactivation is not sufficient since this inactivates a lot more proteins than components of the complement system.

The new experiments suggested by this reviewer have been performed and are presented in the main Figure 2 (for P. aeruginosa strains) and in Supplementary Figure 4 (for other species). Together, those experiments allowed us to address the major but not exclusive role of the complement.

Part II – Major Issues: Key Experiments Required for Acceptance

They should show that the bacteria indeed persist complement and not other heat-sensitive bactericidal components in serum

In order to address this very interesting concern, we used previously described specific complement inhibitor OmCI and assessed the bacterial survival in plasma in the presence of the inhibitor. OmCI inhibits the cleavage of C5 thus preventing MAC formation. The new results are presented in the main Figure 2 (for P. aeruginosa strains) and in Supplementary Figure 4 (for other species). The results of these experiments support our conclusion that the evaders resist the complement mediated killing. Accordingly, we modified the main text and cited the new references on the use of OmCI, lines 169-177, 252-260, 471-484.

Other major issues:
The term persister-like that is used in the title is not fitting that well, since according to the conclusion complement evaders behave differently.

It was difficult to decide whether we should consider Reviewers' 2 or 3 comments as they are completely opposite. Indeed, the Reviewer 3 believes that the persisters and evaders are ***the same*** *sub-populations. This could make sense regarding the diversity of persisters, which have been described in the literature; we therefore "soften" our conclusions that these populations are different, line 262*

We also propose to slightly change the title to: ''Bacterial behavior in human blood reveals complement evaders with ***some*** *persister-like features''. Also, one of the sub-titles where we described growth characteristics of evaders have been changed to read:* ***Evaders are not dormant cells,*** *line 262*

In figure 2, the percentage of surviving cells is higher (10-100 fold) for the bacteria CLJ1, IHMA and PA14 in whole blood compared to plasma. How do the authors explain this difference? Is the number of complement evaders different in whole blood? Is this an effect of the cells that are present?

This is indeed an interesting observation, that we discussed in the main text lines 199-204. Similar behavior was already observed with Salmonella typhimurium (MacLennan 2008), but no hypothesis was given. It is possible that the selective pressure due to complement activity is lower in blood than in plasma, because of all the negative-complement regulators expressed at the surface of circulating leukocytes (i.e CD59, CR1, DAF) but absent in plasma. If the complement activity is lower, then the survival rate will increase. However, other hypothesis are possible.

In figure 2B, additional controls are required to claim that the observed effect is dependent on killing by the membrane attack complex. Since plasma contains a lot of proteins that can have an effect on the survival of bacteria it is necessary to add specific MAC inhibitors (OmCI, Eculizumab). Heat inactivation is not sufficient since this inactivates a lot more proteins than components of the complement system.

Please, see our response to the major (required experiment) comment. OmCI was obtained and was used to inhibit complement-mediated killing in plasma. The new results are presented in the main Figure 2 (for P. aeruginosa strains) and in Supplementary Figure 4 in the revised manuscript.

Figure S2 is in its current form not informative. The authors should include a cell death stain marker to determine if there is a cytotoxic effect. In addition, percentage of CD45 cells should be changed. Quantification of the percentage of dead (purified) neutrophils or PBMCs would be more informative. In the current graphs, the counts of both populations can change and thereby affecting the percentage which makes it hard to interpret the data. Furthermore, in the treated samples events with a lower forward and side scatter are partly outside the gate, that is used for analysis. The cytotoxic data are not required for the manuscript, since the focus is complement evaders.

As proposed and to improve the clarity of these experiments, we assessed the cytotoxic potential of the seven strains of interest on purified PMNs and PBMC, by monitoring LDH release, a reliable marker of cell death (new Fig S2). We believe that even though the cytotoxic potential is indeed not central to this study, this information is of interest as it might allow complement-resistant strains to prevent their elimination by circulating phagocytes (this is what we observed for YIK).

In Figure 3B, the timepoints differ, for some strains there is a timepoint at 1 hour and for some after 2 hours. With the current graph it is hard to judge if there is a biphasic killing curve. Therefore, a few more timepoints and one after 6 hours would be informative to judge if reexposures of the complement evaders result in the same graph.

We believe that the reviewer refers to the point at 2h for the strain CLJ1, where there was no measurable values. The 2h-point was assessed but no colonies could be detected. To enhance the clarity of such cases, we indicated ''nd'' for "not detected" on all graphs. This has been done throughout the manuscript. We also included in the legend of figures with box plots that when the dots are on the X-axis, this means that no colonies were detected (e.g lines 595-596). The main message of Fig 3B is to differentiate between evaders being mutants or phenotypic variants with transient tolerance; kinetic survival during three hours was sufficient to demonstrate that they generated a mainly sensitive population; we therefore do not think that adding more time points will provide more information.

The authors should check that complement activation is similar on stationary and exponential phase bacteria. The expression of LPS O-antigen, capsule and outer membrane proteins changes which are important in complement activation. This is important in the comparison of the number of evaders between exponential and stationary phase. An alternative explanation could be that complement is less well activated on stationary vs exponential phase bacteria.

The molecular mechanisms of bacterial persistence in blood by evaders are currently being addressed in our laboratory. Both the binding of C3b and C5-9 at the bacterial surface, as well as the level of complement activation will be examined in different growth conditions and in different mutants to address this important issue. From our preliminary results obtained from Tn-seq experiments on several P. aeruginosa strains, the expression of many bacterial factors impacts the complement evasion (LPS, alginates, Psl/Pel, and others...).

The complement evaders survive in plasma for a prolonged time. This study would benefit by analyzing complement activation on the evader cells. For instance, check the C3b, C5b-9 levels of the surviving cells by flow cytometry (after 3-6 hours). Is the complement activation and membrane damage the same, but can the evaders deal with the membrane damage in contrast to normal cells?

We agree with the reviewer that the evaders require further characterization, which is underway in our laboratory. Important step in their characterization is the mechanisms underlying evaders' tolerance toward complement-mediated lysis. We believe that evaders are able to escape binding of C3b at their surface; otherwise, they would be efficiently phagocytosed and destroyed in whole blood, which is not the case (now stated in lines 405-410). We thus plan to assess binding of complement factors (C3b, C5-9) at their surface, but this will be done in parallel with evaders' sorting by FACS and subsequent RNA-Seq to assess the transcriptome of these cells (do they over-express known complement-resistance factors?). The experiment proposed here is thus highly relevant, but beyond the scope of this manuscript.

In figure 4B the effect of CCCP indicates that metabolic activity is required for complement evaders. CCCP itself does not kill untreated cells. The data indeed point to the direction that a membrane potential is required. Were the control cells also treated with 0.5 % DMSO, because

this was not clear in the legend and methods. This would be important since DMSO can also affect bacteria. In addition, the statement that metabolic activity is required for complement evaders is only based on the CCCP result. Alternative approaches would help to convince that metabolic activity is required and that this is different compared to persisters (e.g. treatment with bacteriostatic antibiotics).

The control experiment with DMSO is now included in the Fig 4b and the legend was clarified (lines 646-648). We do not see the point of trying with other bacteriostatic agents, as the information obtained with CCCP are clear.

Part III – Minor Issues: Editorial and Data Presentation Modifications

Reviewer #2: Line 54 and 340 "while genetically identical" and "do not harbor genetic mutations"
With this claim you expect that the surviving bacteria (complement evaders) were sequenced and are identical to the original strain. Please add the data or state that this is assumed because reexposure to plasma of the evaders result in a similar percentage of survivors as observed after the initial plasma treatment.

We suppressed these statements, as evaders were not sequenced.

Line 167, hypersensitive change to sensitive

OK, line 166

Figures S1 does not add much information to the manuscript.

Although not central to the manuscript, the cytokine response in blood comforts the idea that all strains are equally well recognized by the immune system. The information is also of interest for colleagues investigating the cellular responses to P. aeruginosa in the blood. Some strains are able to inhibit NF-kB activation in epithelial cells, but the ability of these isolates to prevent cytokine release by circulating leukocytes has never been studied. We would like to keep these results in the study.

Add a detection limit in the graphs of % survival, it is now unclear when there are no surviving cells at all. Sometimes these points are just removed like for PaG8 in figure 5B. Please add this to all graphs.

Please, refer to our previous answer to the concern about Fig 3B. We added information on the graphs and in all legends when needed.

Figure 2C significance is not correctly indicated, should be significant between control and cytochalasin D

Thank you for spotting this mistake!

Legend of figure 3, description jumps from A to C.

The legend was re-organized, lines 633-634.

Line 321-323 "to fully resist in whole blood strains had to display cytotoxicity and complement resistance. This statement is only based on a single strain (YIK), which is not analyzed in detail. More strains and detailed analysis should be performed for this claim.

With the new experiment of cytotoxicity on purified neutrophils, it is clear that YIK kills the entire PMNs population and is the most cytotoxic. Our hypothesis is thus valid, but we attenuated our claim and informed that this should be further assessed for other blood-resistant isolates (lines 343 and 347-348).

It is for some experiments not clear how the bacteria were cultured (only stated exponential phase). Overnight bacteria were diluted and shaken until OD600. Please add some more details, diluted x times in.. at 37C. How is the number of bacteria quantified (since 2.25 E7 bacs/ml are used)?

We added some details in the Methods section, line 433

Figures S3A, it is not clear how this is performed and would be important to compare the bactericidal activity of plasma after 3 hours incubation to fresh plasma. The other option would be to assay the bactericidal activity with a classical complement assay (e.g. CH50)

The goal of the experiment was to assess if the plateau of evaders (well visible at 3h, Fig 3A) is due to a depletion in complement activity rather than presence of a minor population of complement-tolerant cells. We added a diagram to clarify how the experiment was performed (new Fig S3). We observed that during 2nd incubation period, >10^7 bacteria were killed within 1h, showing that the once-used plasma maintained bactericidal activity, showing that the evaders are indeed complement-tolerant. CH50 is an indirect way to assess bactericidal activity, less reliable than directly assessing the survival of the bacteria.

Figure S3B. It would be informative to test the influence of the bacterial concentration over a wider range.

As discussed for reusing the plasma in 2nd incubation, the use of 10 times less bacteria than the usual inoculum comforts our conclusion that complement activity is not limiting.

Line 308-310, statement about exotoxins. The authors did not check if the toxins were expressed and did not test a panel of strains with these toxins. Based on the data shown it is hard to determine that the presence of toxins is unrelated to the strains capacity to survive in blood.

The six P. aeruginosa strains CLJ1, IHMA, PA7, PAO1, PA14 and YIK have all already been characterized in previous studies, and their ability to secrete either ExlA, ExoS or ExoU have been confirmed (Elabbadi et al., 2019; Reboud et al., 2016). Fig 1B recapitulates the nature of the toxin secreted by each strain.

Line 345-346 describes the complement resistant *K. pneumoniae*, would be informative if this comes back in the discussion. Was this selection of a subpopulation (e.g. heteroresistant) or probably a mutation that causes this different phenotype?

Upon regrowth, one out of three K. pneumonia colonies gave rise to a fully resistant population. If this was heteroresistance (unstable phenotype), the progeny would have been as sensitive as the parental one, which is not the case. The selection of the mutant is hypothesized in the main text, lines 248-251

Legend fig S5 – Check description, it states plating for 2 hours, should be incubation.

We revised this sentence.

Reviewer #3:

Part I - Summary

This study seeks to better understand the ability of the bacteria, Pseudomonas aeruginosa, to survive and cause serious infection in the human blood stream. To do this, they assay the ability of six strains (3 lab strains, & 3 clinically isolated strains) to survive in human whole blood (HWB), revealing significant variability between and within strains in the ability to survive complementation. These differences were not accounted for by genetic differences and so the study has the potential to make an important contribution to our understanding of our particular vulnerability to this kind of infection, and phenotypic defence mechanisms of bacteria more generally.

A subpopulation of survivors was apparent in all of the tested P. aeruginosa strains leading the authors to speculate that these HWB 'evaders' may be similar to bacterial persisters (defined in the literature as phenotypic variants within a bacterial population that are able to transiently survive high antibiotic doses, while remaining genetically identical to other cells within the population). To explore the potential role of persistence in HWB-treated survival, the authors conducted a series of killing curves to test for the expected biphasic killing curves, as seen with persister cells within the literature. The killing curves showed that all strains showed similar phenotypic variation in response to HWB.

After detecting complement evaders in all P. aeruginosa strains, the authors expanded their assay to include 7 further species of Gram-negative bacteria. Two species demonstrated non-biphasic killing curves in HWB, however, the remaining five showed distinct killing curves.

To further explore the potential for 'evaders' to be persister cells, the authors carried out assays on log versus stationary growing cells, since the literature has highlighted that persister cells predominantly emerge during stationary phase. Contrary to expectation given the literature, three P. aeruginosa strains and two of the selected Gram-negative species generated a greater proportion of 'evaders' in exponential phase. Studies already conducted on persister formation have highlighted the roll of cell dormancy in antibiotic survival. Therefore, the authors explored the role of inhibiting metabolic activity with varying results. Lastly, the different strains and species were observed by microscopy to evaluate division rates after incubation with plasma and heat-inactivated plasma. No significant difference was observed between the heat-treated/active plasma growth rates. Taken together, the authors deduced that metabolism played a key role in 'evader' formation.

Strength: this is a detailed and fascinating study of a clinically important yet under-studied bacterial behaviour. The paper is clearly written and easy to follow (thank you!)

Weakness: evidence for evasion being a distinct behaviour from persistence was not so convincing and not necessary for the paper to make an important contribution.

Part II – Major Issues: Key Experiments Required for Acceptance

We (I am writing this review with my PhD student, so we are a reviewer team) were concerned that the evidence for evaders to be difference from persister cells was interpreted too strongly, especially in the light of continuing controversy over what persisters really are and how to categorise them. (The importance of the paper's potential contribution does not rest on there being a distinction in our view.)

We, therefore suggest the claim for them to be different is qualified in light of recent literature: there are perhaps too many similarities between persisters and 'evaders' for 'evaders' to be

defined as a distinct physiological state. Balaban et al., 2019 explore the notion of spontaneous versus triggered persisters. There is some indication that spontaneous persisters arise during exponential growth, therefore, we may expect metabolism to affect persister proportion during exponential growth. The distinct complement evasion strategy is an interesting notion within this study, though it should be explored further before any distinction between persisters and 'evaders' can be made. There is perhaps scope to assay the strains with a common antibiotic to compare with HWB.

We agree with the concern and thank to both reviewers for the constructive remarks and point of view. Indeed, it is likely that there are as many (or more) "persister states" (or ways to survive) as there are antimicrobials (or ways to kill). We therefore revised our comparison with antibiotic persisters (e.g lines 34-38, 262 or 365-366), and used Figure 4 to better characterize these cells, without the aim of differentiating them necessary from antibiotic-persisters. We conclude that the evaders allow bacterial persistence in the blood, but not to confuse them with persisters to antibiotics, we purposely gave them the different name.

<u>Part III – Minor Issues: Editorial and Data Presentation Modifications</u>

Lines 479-481: On average, how many cells were observed? Were there only two replicates for each strain and/or species? This will affect how reliable the observations were as at least three replicates should have been used

Altogether, eight individual cells on the pad were tracked for each condition (normal plasma and heat-inactivated plasma) in two independent experiments. Therefore, the data represent 16 independent events.

Line 491: 'depending of the normality' should be 'depending on…'

OK

Figure 2: The patterns and data points and colours used on the graph are a bit nasty to look at. The added data point circles impair boxplot visualisation.

We reduced the size of point circles to increase the clarity.

Figure 3: The order of the lettering in the legend is unclear. 'A)' goes to 'C)', which then goes to 'B) and D)'. Could these figures be re-ordered, or the legend order altered?

The legend was re-organized, lines 633-634.
